# The roles of exosomal miRNAs and lncRNAs in lung diseases

**DOI:** 10.1038/s41392-019-0080-7

**Published:** 2019-11-13

**Authors:** Yang Li, Zhengrong Yin, Jinshuo Fan, Siyu Zhang, Weibing Yang

**Affiliations:** Key Laboratory of Respiratory Diseases of the Ministry of Health, Department of Respiratory and Critical Care Medicine, Union Hospital, Tongji Medical College, Huazhong University of Science and Technology, 1277 Jiefang Avenue, 430022 Wuhan, China

**Keywords:** Respiratory tract diseases, Predictive markers, Non-coding RNAs

## Abstract

An increasing number of studies have reported that exosomes released from various cells can serve as mediators of information exchange between different cells. With further exploration of exosome content, a more accurate molecular mechanism involved in the process of cell-to-cell communication has been revealed; specifically, microRNAs (miRNAs) and long noncoding RNAs (lncRNAs) are shuttled by exosomes. In addition, exosomal miRNAs and lncRNAs may play vital roles in the pathogenesis of several respiratory diseases, such as chronic obstructive pulmonary disease (COPD), lung cancer, and asthma. Consequently, exosomal miRNAs and lncRNAs show promise as diagnostic biomarkers and therapeutic targets in several lung diseases. This review will summarize recent knowledge about the roles of exosomal miRNAs and lncRNAs in lung diseases, which has shed light on the discovery of novel diagnostic methods and treatments for these disorders. Because there is almost no published literature about exosomal lncRNAs in COPD, asthma, interstitial lung disease, or tuberculosis, we summarize the roles of exosomal lncRNAs only in lung cancer in the second section. This may inspire some new ideas for researchers who are interested in whether lncRNAs shuttled by exosomes may play roles in other lung diseases.

## Introduction

Extracellular vesicles (EVs) are small vesicles shed or released from cell membranes.^1^ According to their size and biogenesis, EVs are divided into apoptotic bodies, ectosomes (microparticles/microvesicles), and exosomes.^2^ Among these EV types, exosomes, with a size of approximately 30–100 nm,^3^ are produced through several steps, including the formation of endosomes, inward budding in these endosomes, causing the biogenesis of multivesicular bodies (MVBs), the fusion of MVBs with the cell membrane, and the release of exosomes outside cells.^4^ Exosomes are present in various bodily fluids, such as the blood,^5,6^ sputum,^7^ and urine,^8^ which can be collected to extract exosomes. It has been demonstrated that exosomes secreted by cells play an indispensable role in intercellular communication^9^ by carrying biomolecules, including proteins, messenger RNAs (mRNAs), DNA, and noncoding RNAs (ncRNAs).^3^ In particular, many studies have indicated that exosomes transferring ncRNAs such as microRNAs (miRNAs) and long ncRNAs (lncRNAs) can influence diverse biological processes in other cells.^10^

In general, ncRNAs are RNA molecules with no ability to be translated into protein.^11^ NcRNAs primarily comprise small ncRNAs and lncRNAs, which are divided on the basis of their length.^12^ Small ncRNAs have fewer than 200 nucleotides (nt), while the lengths of lncRNAs are over 200 nt.^13^ Moreover, small ncRNAs can be further categorized into miRNAs, PIWI-interacting RNAs (piRNAs) and small interfering RNAs (siRNAs),^11^ among which miRNAs have been widely investigated. NcRNAs, especially miRNAs and lncRNAs, can regulate gene expression through distinct mechanisms.^14^ For instance, miRNAs modulate gene expression at the post-transcriptional level by binding to the 3′ noncoding regions of their target mRNAs.^15^ LncRNAs can be involved in post-transcriptional and transcriptional regulation.^16^ In addition, lncRNAs can affect mRNA translation by serving as miRNA sponges,^17^ which can combine with specific miRNAs to suppress the function of the target miRNAs and thereby regulate the expression of downstream target genes.^18^ To some extent, extracellular miRNAs and lncRNAs remain stable partly due to the protection afforded by exosomes.^19^ Moreover, miRNAs and lncRNAs in exosomes released by donor cells can be taken up by recipient cells and modulate gene expression in these cells.^20^ However, the expression patterns of miRNAs and lncRNAs vary under different physiological and pathological conditions, which may mean that these exosomal biomolecules have the potential to reflect disease states.^21^

In fact, numerous studies have reported the functions of exosomal miRNAs or lncRNAs in diseases of different systems, including cardiovascular,^22,23^ neurological,^24,25^ and urinary tract^26,27^ diseases, especially malignant tumors^28,29^ in these systems. Similar progress has been made in respiratory diseases. Emerging studies have shown that exosomal miRNAs and lncRNAs play important roles in the pathogenesis and progression of several lung diseases, including lung cancer, chronic obstructive pulmonary disease (COPD), asthma, tuberculosis (TB), and interstitial lung disease (ILD).^30,31^ In addition, many studies have reported that the exosomal miRNA or lncRNA profiles in patients with lung disease differ from those in healthy people.^32–34^ Thus, exosomal miRNAs and lncRNAs seem to have the potential to become noninvasive diagnostic biomarkers and therapeutic targets in pulmonary diseases. It is essential to comprehensively summarize the latest studies about the utility of exosomal miRNAs and lncRNAs in pulmonary diseases. In this review, we will highlight the functional roles of exosomal miRNAs and lncRNAs in the pathogenesis, diagnosis, and therapy of lung diseases.

## Exosomal miRNAs in lung diseases

To date, studies have mainly focused on lung cancer in regard to the roles of exosomal miRNAs in lung diseases. Thus, in the first section, our review largely concentrates on several aspects of the roles of exosomal miRNAs in lung cancer, and we present only an overview of exosomal miRNAs in other lung diseases.

### Exosomal miRNAs in COPD

COPD is mainly characterized by airway reconstruction as well as irreversible and progressive airflow limitation^35^ resulting from infection,^36^ cigarette smoke extract (CSE),^37^ or other detrimental stimuli. How these irritating factors contribute to the development of airway reconstruction and airflow limitation is still not fully understood.^38^ However, recent advances in studies concerning exosomes and exosomal miRNAs may shed new light on potential mechanisms. The characteristic exosomal miRNAs in these mechanisms may act as diagnostic biomarkers and therapeutic targets for COPD.

Since the lungs connect to the external environment through the airways, bronchial and alveolar epithelial cells act as significant components of the first line of the host immune defense.^39^ Upon exposure to various stimuli, these constitutively exposed cells may become injured and secrete exosomes containing specific miRNAs into the extracellular space^40^ to facilitate the recovery of epithelial cells from injury by activating static stem cells and epithelial cells and restoring the cell cycle in these cells.^41^ Moreover, exosomes secreted by these injured or stimulated cells can also trigger a series of inflammatory responses that promote the clearance of harmful stimuli and lead to the pathogenesis of some inflammation-related diseases, such as COPD. Thus, exosomes can maintain the physiological balance of the local microenvironment by clearing harmful stressors and promoting tissue repair as well as result in the development of certain diseases, such as COPD.^42^ Among these processes, specific exosomal miRNAs play a crucial role in influencing the functions of other cells, such as other epithelial cells that are not exposed to these harmful stressors.^43^ Some studies have demonstrated that stressors, regardless of their endogenous or exogenous origin, can promote exosome release. For example, Benedikter et al.^44^ revealed that CSE exposure could boost the number of exosomes secreted by bronchial epithelial cells (BEAS-2B cells). Stressors such as heat shock, hypoxia, and oxidant exposure can also increase the secretion of EVs, such as exosomes, and change their cargo, including miRNAs.^45^ Tan et al.^46^ found that exosomes in the plasma from acute exacerbation COPD (AECOPD) and stable COPD (sCOPD) patients outnumbered those in the plasma from healthy subjects and that the highest numbers of plasma exosomes were found in AECOPD patients. In addition, their results supported the conclusion that the exosome level was associated with the levels of some inflammatory factors, such as C-reactive protein, soluble tumor necrosis factor-α (TNF-α) receptor-1, and interleukin-6 (IL-6), in the plasma, which implied a possible role for exosomes in the inflammatory reactions of COPD. Since smoking and infection are the most common causes of COPD and AECOPD, respectively, and function in inducing exosome secretion, it is reasonable that patients with AECOPD show a higher level of plasma exosomes than patients with sCOPD and that there is an elevated plasma exosome level in sCOPD patients compared with healthy controls. Considering the correlations of plasma exosome levels with certain inflammatory factors, exosomes can be used as indicators to monitor the progression of COPD and reducing exosome levels may be a new approach for COPD therapy. Obviously, whether these hypotheses can be put into practice requires further studies regarding the concrete mechanism underlying the involvement of exosomes in the inflammatory reaction of COPD.

**Fig. 1:**
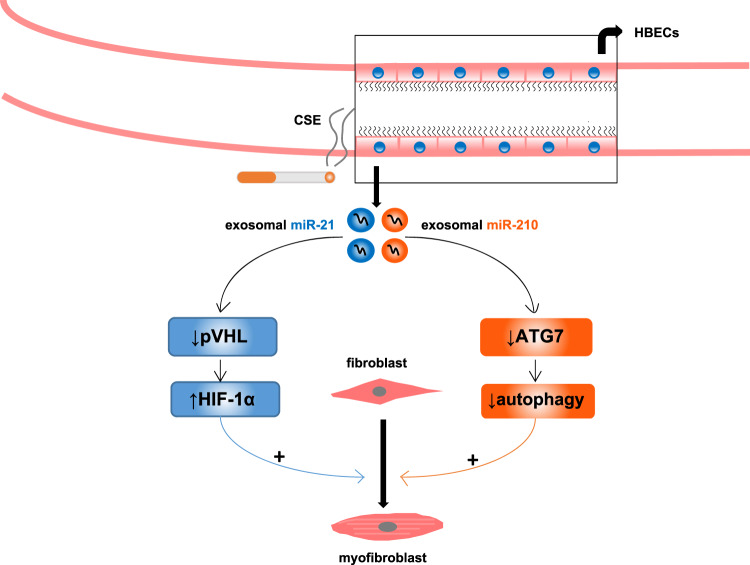
The role of exosomal miRNAs in abnormal information exchange between the bronchial epithelium and bronchial fibroblasts, which results in the differentiation of fibroblasts into myofibroblasts. ↓: downregulate or reduce, ↑: upregulate, +: promote

Moreover, stimuli such as CSE can not only influence the levels of exosomes but also change the expression of specific miRNAs in exosomes. For instance, one study reported that smoking altered the miRNA profile of EVs, including exosomes, in the bronchoalveolar lavage fluid (BALF) by comparing the profiles in smokers and nonsmokers.^38^ A recent study also indicated that CSE could modify exosome compositions. It was discovered that the levels of exosomal *miR-21* from human bronchial epithelial cells (HBECs) exposed to CSE were higher than those from HBECs without CSE treatment.^47^ Several studies have shown that abnormal information exchange between the bronchial epithelium and bronchial fibroblasts mediated by specific exosomal miRNAs results in the differentiation of fibroblasts into myofibroblasts through specific mechanisms (Fig. 1). In addition, it has been reported that myofibroblasts originating from fibroblasts can lead to airway remodeling by producing extracellular matrix components such as collagenous proteins and α-smooth muscle actin (α-SMA), which endows myofibroblasts with strong contractile activity.^48,49^ Fujita et al.^50^ reported that the upregulated *miR-210* level in CSE-induced EVs, such as exosomes from HBECs, could boost the transformation of lung fibroblasts (LFs) into myofibroblasts by targeting autophagy-related 7 (*ATG7*), as insufficient *ATG7* expression would lead to a decrease in autophagy, whose insufficiency would lead to myofibroblast differentiation from LFs. Furthermore, they found that CSE-induced EV *miR-210* from HBECs could significantly increase the expression of α-SMA and collagen type I in LFs and enhance myofibroblast differentiation. These findings are in agreement with those of another study that indicated that exosomal *miR-21* from CSE-treated HBECs could promote the differentiation of fibroblasts into myofibroblasts by targeting von Hippel–Lindau protein (*pVHL*). Moreover, the downregulation of *pVHL* expression can increase the protein levels of α-SMA and collagen I by facilitating the expression of hypoxia-inducible factor 1α (*HIF-1α*).^47^ These described mechanisms in combination with the function of myofibroblast differentiation in airway remodeling may provide novel clues for the diagnosis and treatment of COPD. In other words, characteristic miRNAs in exosomes from patients with COPD may act as diagnostic biomarkers, and adjustments to these exosomal miRNAs or their downstream targets may serve as therapeutic approaches. In addition, some investigators have suggested that eliminating exosomes that contain specific miRNAs could be a promising therapeutic method in COPD,^51^ and this suggestion is consistent with our preceding analysis that indicates that decreasing exosome levels may become a therapeutic tool.

### Exosomal miRNAs in asthma

Asthma is a class of chronic inflammatory diseases caused by nonspecific stimuli in the airways with the involvement of various cells, including T lymphocytes, mast cells, eosinophils, neutrophils, and airway epithelial cells.^52^ Airway hyperresponsiveness (AHR), airway inflammation, and airway remodeling are the main factors in the pathogenesis of asthma.^53^ Emerging evidence shows that exosomes or exosomal miRNAs released by asthma-associated cells, such as mast cells,^54^ eosinophils,^55^ neutrophils,^56^ and T lymphocytes,^57^ can function as mediators of intercellular information exchange, thereby contributing to AHR, airway inflammation, and airway remodeling.^58^ It was proven that exosomal miRNAs from human mast cells could be shuttled to other mast cells and participate in intercellular crosstalk.^59^ An investigation by Mazzeo et al.^60^ observed that MVBs in eosinophils from asthmatic patients had the ability to release higher levels of exosomes than those from healthy people. Another study further explored the hypothesis that exosomes derived from eosinophils in patients with asthma could promote apoptosis in small airway epithelial cells and proliferation in bronchial smooth muscle cells, which were responsible for airway remodeling.^61^ Moreover, lipopolysaccharide-induced exosomes from neutrophils were also reported to have the ability to increase airway smooth muscle (ASM) cell proliferation, which plays an essential role in airway remodeling in asthma.^56^ Kulshreshtha et al.^62^ discovered that exosomes from IL-13-treated BEAS-2B cells were capable of enhancing the proliferation and chemotaxis of monocytes involved in airway inflammation. In addition, they also found that the use of the exosome suppressor GW4869 could relieve certain asthmatic manifestations, such as methacholine-induced AHR, inflammation, and mucus production.^62^ However, none of the results described above identified the particular miRNAs in exosomes that were important in these processes.

To identify the miRNAs in exosomes that participate in the development of asthma, some studies have measured exosomal miRNA profiles in asthmatic and healthy groups (Table 1). A previous study confirmed eight exosomal miRNAs (*let-7a*, *miRNA-21*, *miRNA-658*, *miRNA-24*, *miRNA-26a*, *miRNA-99a*, *miRNA-200c*, and *miRNA-1268*) with substantial expression differences in the BALF between healthy subjects and patients with mild intermittent asthma.^33^ Similarly, an animal model experiment indicated that some miRNAs (*miR-1827*, *miR-346*, and *miR-574-5p*) had consistently upregulated expression in EVs, such as exosomes, secreted by airway cells in mice with asthma compared with sham-control mice via both microarray and quantitative reverse transcription-polymerase chain reaction (qRT-PCR) analyses. In addition, GW4869, an inhibitor of exosome generation, could reduce the levels of EVs, T-helper type 2 cytokines and eosinophils in the BALF and suppress eosinophil deposition in the airway walls and mucosa, which indicated that decreasing exosomes could relieve allergic airway inflammation.^63^ However, the authors did not further verify whether these altered exosomal miRNAs played a crucial role in allergic airway inflammation. In a rat study, 23 serum exosomal miRNAs with differential expression between rats with airway inflammation caused by zinc oxide nanoparticles and controls were identified. According to functional and pathway analyses, these exosomal miRNAs with differential expression might be involved in pulmonary inflammation, but this kind of prediction still requires further validation via experiments.^64^ Nonetheless, it remains unclear how these exosomal miRNAs with differential expression contribute to the pathology of asthma. It has been reported that respiratory syncytial virus (RSV) and rhinovirus (RV) are significant factors contributing to the exacerbation and development of asthma.^65^ Specific miRNAs produced by airway epithelial cells (AECs) and peripheral blood mononuclear cells result in AHR by regulating the activity of leukotrienes; influencing the function, differentiation, and proliferation of immune cells; and modulating the immunological reaction after RSV infection.^66^ Gutierrez et al.^67^ characterized the profiles of airway secretary miRNAs by measuring the expression of nasal exosomal miRNAs in children with or without RV infection. They identified four exosomal miRNAs (*hsa-miR-630*, *hsa-miR-302d-3p*, *hsa-miR-320e*, and *hsa-miR-612*) that were constitutively expressed in nasal airway secretions in both the RV infection and control groups and found that exosomal *miR-155* was detectable only in children with RV infection.

**Table 1:** miRNA profiles in exosomes from an asthma group vs. those from a control group

Groups	Bodily fluids	Exosomal miRNA with altered expression	References
Asthma vs. healthy control (human)	BALF	Let-7a, miR-21, miR-658, miR-24, miR-26a, miR-99a, miR-200c, miR-1268.	Levanen et al.^33^
Asthma vs. healthy control (mice)	BALF	miR-1827, miR-346, miR-574-5p.	Gon et al.^63^

Despite extensive investigations regarding the roles of miRNAs in the development of asthma,^68^ there is still a lack of exploration into how exosomal miRNAs function in this disease. Therefore, to develop effective therapeutic strategies and accurate diagnostic methods for asthma, it is necessary to clarify the potential mechanisms involved, such as the target genes that these exosomal miRNAs regulate and the asthma-related signaling pathways that these target genes control.

### Exosomal miRNAs in ILD

ILD is a general term for a group of lung disorders^69^ with diffuse pulmonary interstitial fibrosis as the main pathological characteristic.^70^ To date, explicit causes have been found only for some of these disorders, while those without a definite cause are named idiopathic interstitial pneumonia (IIP).^71^ Idiopathic pulmonary fibrosis (IPF) is regarded as the most common^72^ and most severe category^73^ of IIP. However, due to its unclear etiology and pathogenesis, there are considerable obstacles to finding exact diagnostic methods and effective treatments for IPF.^74^ As there are more studies concerning the roles of exosomes and exosomal miRNAs in various cancers, such as lung cancer,^75^ and there are some common epigenetic mechanisms between cancer and IPF,^76^ it is rational to explore whether these new kinds of biomarkers participate in the pathogenesis of IPF. Njock et al.^34^ reported significant differences in exosomal miRNA profiles in the sputum between patients with IPF and healthy subjects. Among these miRNAs with differential expression, researchers identified two miRNAs (*miR-142-3p* and *miR-33a-5p*) with upregulated expression and one miRNA (*let-7d-5p*) with downregulated expression as biomarkers and reported a higher area under the curve (AUC, 0.978) when combining these three sputum-derived exosomal miRNAs with model 1 (a logistic regression model for the diagnosis of IPF) than when using model 1 alone. Makiguchi et al.^77^ analyzed the miRNA profiles in serum EVs, including exosomes, and found that *miR-21-5p* in serum EVs was significantly upregulated in patients with IPF compared with healthy controls. In addition, there was a significant correlation between the level of serum EV *miR-21-5p* and mortality in IPF patients during the 30-month follow-up, and those with higher expression of serum EV *miR-21-5p* had a poorer prognosis. Thus, *miR-21-5p* in serum EVs, including exosomes, could act as a promising prognostic biomarker in IPF. The results of an animal experiment indicated a decrease in *miR-29c* expression in alveolar epithelial type II cells from mouse lungs with IPF compared with those from healthy mouse lungs and that *miR-29c* had the ability to maintain epithelial integrity, facilitate recovery from injury, and decrease lung fibrosis in mice.^78^ Furthermore, it has been reported that exosomes can act as beneficial carriers of molecules such as miRNAs.^79^ Hence, including beneficial miRNAs such as *miR-29c* in exosomes may be a novel mode of targeted therapy for IPF.

### Exosomal miRNAs in TB

TB is a contagious lung disease caused by *Mycobacterium tuberculosis* (Mtb).^80^ Mtb that infects the human body survives in host cells, especially macrophages, if not removed by the immune defense system of the host.^81^ Therefore, the interaction between Mtb and host immune cells, such as macrophages, may affect the pathogenesis of TB.^82^ In fact, it has been revealed that macrophages infected with Mtb can release particular miRNAs packaged in exosomes into the extracellular space. An example can be taken from a study that proved that monocyte-derived macrophages (MDMs) infected by *Mycobacterium bovis* bacillus Calmette–Guérin (BCG) were able to release specific exosomal miRNAs, including *miR-1224*, *miR-1293*, *miR-425*, *miR-4467*, *miR-4732*, *miR-484*, *miR-5094*, *miR-6848*, *miR-6849*, *miR-4488*, and *miR-96*.^83^ Singh et al.^84^ discovered that Mtb infection in macrophages could suppress the encapsulation of specific miRNAs in exosomes and that these miRNAs appeared to regulate target genes related to immune surveillance as well as inflammation. It was also reported that exosomes from macrophages infected by Mtb contained a set of unique host miRNAs and mycobacterial RNAs that played roles in the process of Mtb infection and served as diagnostic biomarkers for TB.

Some studies have explored the utility of blood exosomal miRNAs as diagnostic biomarkers for TB. Based on their previous research, Alipoor et al.^85^ chose 3 of the 11 exosomal miRNAs with the highest expression (*miR-484*, *miR-425*, and *miR-96*) as diagnostic biomarkers to evaluate their diagnostic value for TB. The receiver operating characteristic (ROC) curve analysis indicated that the AUC values of serum exosomal *miR-484*, *miR-425*, and *miR-96* were 0.72, 0.66, and 0.62, respectively, and it was also found that the expression levels of the three serum exosomal miRNAs were closely related to the extent of TB infection, which was defined according to the grade of smear positivity. Similarly, a study by Lyu et al.^86^ compared the profiles of serum exosomal miRNAs among patients with latent TB infection (LTBI), patients with active TB (ATB) infection and healthy individuals, and their results showed that four exosomal miRNAs (*hsa-let-7e-5p*, *hsa-let-7d-5p*, *hsa-miR-450a-5p*, and *hsa-miR-140-5p*) were specifically expressed in the LTBI group, while five exosomal miRNAs (*hsa-miR-1246*, *hsa-miR-2110*, *hsa-miR-370-3P*, *hsa-miR-28-3p*, and *hsa-miR-193b-5p*) were specific to ATB infection. Their study revealed that serum exosomal miRNAs had the potential to act as diagnostic biomarkers for LTBI and ATB infection. Another study explored the diagnostic value of combining exosomal miRNAs with electronic health records (EHRs) in TB. They found that six plasma exosomal miRNAs *(miR-20a, miR-20b, miR-26a, miR-106a, miR-191*, and *miR-486)* showed significant expression differences between TB patients and controls. The model that combined exosomal miRNAs with EHRs showed the best diagnostic power, with an AUC of 0.97 for both pulmonary TB and TB meningitis.^87^

Pleural effusion (PE) is one of the major manifestations of TB when the infection spreads to the pleura and can be extracted to analyze its components and distinguish TB from other lung diseases, such as lung cancer and pneumonia.^88^ A previous study explored the differences in PE exosomal miRNA profiles among lung adenocarcinoma (LAC), TB, and other benign lesions via deep sequencing and qRT-PCR. There were three miRNAs (*miR-148a-3p*, *miR-451a*, and *miR-150-5p*) with differential PE expression between the TB group and the other benign lesions group, while nine exosomal miRNAs (*miR-205-5p*, *miR-483-5p*, *miR-375*, *miR-200c-3p*, *miR-429*, *miR-200b-3p*, *miR-200a-3p*, *miR-203a-3p*, and *miR-141-3p*) in the PE samples were detected to have significant differences between the LAC group and the other two groups.^89^

Given the potential of these differentially expressed exosomal miRNAs as diagnostic biomarkers for TB (Table 2), further investigations should be carried out to clarify the mechanisms by which these exosomal miRNAs contribute to the pathogenesis of TB, especially in the interaction between Mtb and immune cells, thereby assisting in the development of novel approaches for the diagnosis and treatment of TB.

**Table 2:** Exosomal miRNAs with differential expression act as diagnostic biomarkers for TB

Groups	Bodily fluids	Exosomal miRNA with different expression	Functions	References
MDMs with vs. without BCG infection	Cell culture	miR-1224, miR-1293, miR-425, miR-4467, miR-4732, miR-484, miR-5094, miR-6848, miR-6849, miR-4488, miR-96	Diagnostic biomarkers	Alipoor et al.^83^
TB vs. HC	Serum	miR-484, miR-425, miR-96	Diagnostic biomarkers	Alipoor et al.^85^
LTBI vs. ATB vs. HC	Serum	hsa-let-7e-5p, hsa-let-7d-5p, hsa-miR-450a-5p, hsa-miR-140-5p (LTBI specific) hsa-miR-1246, hsa-miR-2110, hsa-miR-370-3P, has -miR-28-3p, hsa-miR-193b -5p (ATB specific)	Diagnostic biomarkers	Lyu et al.^86^
TB vs. controls	Plasma	miR-20a, miR-20b, miR-26a, miR-106a, miR-191, miR-486	Diagnostic biomarkers	Hu et al.^87^
APE vs. TPE vs. NPE	PE	miR-148a-3p, miR-451a, miR-150-5p (TPE vs. NPE) miR-205-5p, miR-483-5p, miR-375, miR-200c-3p, miR-429, miR-200b-3p, miR-200a-3p, miR-203a-3p, miR-141-3p (APE vs. TPE and NPE)	Diagnostic biomarkers	Wang et al.^89^

*HC* healthy control, *APE* lung adenocarcinoma with pleural effusion, *TPE* tuberculous with pleural effusion, *NPE* other benign lesions with pleural effusion

### Exosomal miRNAs in lung cancer

Lung cancer is a malignant neoplastic disease that is considered to be the primary cause of cancer-related death.^90^ Thus, an increasing number of researchers are working to clarify the mechanisms of its occurrence and progression as the basis for developing more valuable diagnostic and therapeutic methods for lung cancer.^91^ Substantial reports have suggested that exosomal miRNAs play vital roles in various cancer-related pathological processes by acting as mediators of communication between lung cancer cells and other cells,^92^ which is undoubtedly a breakthrough toward the discovery of novel biomarkers and therapeutic targets in lung cancer.

### Exosomal miRNAs as diagnostic and prognostic biomarkers

Some studies have compared exosomal miRNA expression in patients with lung cancer and healthy subjects (Table 3). For instance, an investigation analyzed the expression of 84 exosomal miRNAs in the plasma from 10 patients with non-small-cell lung cancer (NSCLC) and 10 healthy people and found 30 miRNAs exhibiting differential expression. In addition, by combining the 30 differentially expressed exosomal miRNAs with relevant results reported in the literature, the researchers selected plasma exosomal *miR-23b-3p*, *miR-10b-5p*, and *miR-21-5p* as prognostic biomarkers for NSCLC and improved the AUC by adding these three exosomal miRNAs to a clinical prognostic variable model.^93^ The three plasma exosomal miRNAs, *miR-19-3p*, *miR-21-5p*, and *miR-221-3p*, exhibited upregulated expression in patients with LAC compared with healthy controls.^94^ Rodriguez et al.^95^ discovered that only exosomal *miR-141* expression was significantly lower in NSCLC patients than in nontumor patients by characterizing the expression of exosomal miRNAs in the plasma of NSCLC and nontumor patients through miRNA quantitative PCR analysis and validation trials. Zhang et al.^96^ found that serum exosomal *miR-17-5P* was expressed at higher levels in NSCLC patients than in healthy individuals and implied that serum exosomal *miR-17-5p* might serve as a diagnostic biomarker for NSCLC. Another study by Cazzoli et al.^32^ further explored the differences in plasma exosomal miRNAs among three groups, namely, an LAC group, a lung granuloma group and a healthy smoker group. Ultimately, they selected four exosomal miRNAs to screen for differences between the nodule groups (LAC and lung granulomas) and the nonnodule group and six exosomal miRNAs for a second test discriminating between LAC and granuloma in the nodule population. Moreover, exosomal miRNAs have also been reported to serve as biomarkers for distinguishing different types of NSCLC. A previous investigation identified four exosomal miRNAs (*miR-181-5p*, *miR-30a-3p*, *miR-30e-3p*, and *miR-361-5p*) and three exosomal miRNAs (*miR-10b-5p*, *miR-15b-5p*, and *miR-320b*) in the plasma that were adenocarcinoma specific and squamous cell carcinoma specific, respectively.^97^ Poroyko et al.^98^ studied patients with NSCLC or small-cell lung cancer (SCLC) and identified 18 or 16 serum exosomal miRNAs with differential expression between NSCLC or SCLC patients, respectively, and healthy subjects.^98^ In brief, exosomal miRNAs, with differential expression between lung cancer patients and healthy controls or patients with other diseases that require a differential diagnosis from lung cancer, can function as significant diagnostic biomarkers of lung cancer. These exosomal miRNAs can be selected for further studies on whether they play important roles in the initiation and progression of lung cancer and underlying mechanisms.

**Table 3:** Exosomal miRNAs with differential expression in blood from patients with lung cancer compared with that from healthy people or nontumor patients, as well as the utilities of the exosomal miRNAs in lung cancer

Groups	Bodily fluids	Exosomal miRNAs	Utilities in lung cancer	References
NSCLC vs. HC	Plasma	miR-23b-3p, miR-10b-5p, miR-21-5p	Biomarkers for prognosis	Liu et al.^93^
LAC vs. HC	Plasma	miR-19-3p, miR-21 -5p, miR-221-3p	Diagnostic biomarkers	Zhou et al.^94^
NSCLC vs. nontumor	Plasma	miR-141	Diagnostic biomarkers	Rodriguez et al.^95^
NSCLC vs. HC	Serum	miR-17-5p	For diagnosing	Zhang et al.^96^
LAC vs. LG vs. HC	Plasma	4 miRNAs	For screening	Cazzoli et al.^32^
		6 miRNAs	For diagnosing	
NSCLC vs. HC	Serum	18 miRNAs	Diagnostic biomarkers	Poroyko et al.^98^
SCLC vs. HC		16 miRNAs		
LAC vs. HC	Plasma	miR-181-5p, miR- 30a-3p, miR-30e-3p, miR-361-5p	Diagnostic biomarkers	Jin et al.^97^
LSCC vs. HC		miR-10b-5p, miR-15b-5p, miR-320b		

*LG* lung granulomas, *LSCC* lung squamous cell carcinoma

### Multiple facets of exosomal miRNAs in lung cancer progression

**Fig. 2:**
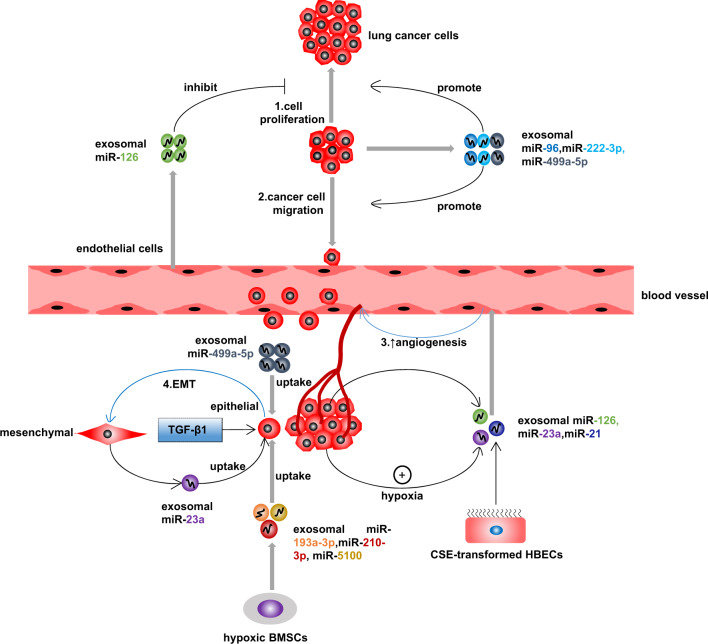
Exosomal miRNAs participate in proliferation, migration, angiogenesis, and EMT in lung cancer cells and therefore promote tumor growth, metastasis, and invasion. ↑ promote; BMSCs bone marrow-derived mesenchymal stem cells

Emerging results show that miRNAs in exosomes are associated with diverse pathological processes, such as the proliferation and migration of lung cancer cells, angiogenesis, and epithelial–mesenchymal transformation (EMT), which contribute to tumor growth, metastasis, and invasion in lung cancer (Fig. 2).

Wu et al.^99^ revealed that the levels of *miR-96* in serum exosomes from patients with lung cancer significantly exceeded those in serum exosomes from healthy people and that *miR-96* was highly expressed in exosomes from highly invasive lung cancer cells. They concluded that circulating exosomal *miR-96* played a promotive role in lung cancer cell proliferation and migration by targeting LIM-domain-only protein 7 (*LMO7*) and that treatment with a *miR-96* inhibitor or the overexpression of *LMO7* could reverse this promotive action. Another study indicated that serum *miR-126* was primarily found in exosomes in NSCLC patients, while the levels of exosomal *miR-126* and exosome-free *miR-126* in the serum were similar in healthy subjects. In addition, it was reported that exosomal *miR-126* from human umbilical vein endothelial cells (HUVECs) was able to inhibit the proliferation of NSCLC cells and decrease their malignancy by targeting insulin receptor substrate 1 (*IRS1*) and vascular endothelial growth factor (*VEGF*).^100^ Another study demonstrated that exosomes could enhance the proliferation and migration of lung cancer cells by transferring *miR-222-3p*, and a correlation analysis revealed that a high level of exosomal *miR-222-3p* in NSCLC patient serum was closely related to a poor prognosis and tumor metastasis after gemcitabine treatment.^101^ He et al.^102^ found higher expression of *miR-499a-5p* in exosomes derived from highly metastatic SPC-A-1BM lung cancer cells than in exosomes derived from SPC-A-1 lung cancer cells, with a lower capacity for metastasis. They proved that in contrast to SPC-A-1 cells, highly expressed exosomal *miR-499a-5p* from SPC-A-1BM cells could contribute to the proliferation and migration of recipient lung cancer cells. In addition, the inhibition of *miR-499a-5p* could suppress this effect, while the overexpression of *miR-499a-5p* could enhance cell proliferation and migration. Fabbri et al.^103^ reported that exosomal *miR-21*, *miR-27b*, and *miR-29a* showed significantly higher expression in NSCLC cells than in normal HEK-293 cells. These results indicate that *miR-21* and *miR-29a* in exosomes derived from lung cancer cells can increase the secretion of inflammatory cytokines such as TNF-α and IL-6 and promote a prometastatic inflammatory response by binding to and activating Toll-like receptors in immune cells.

Angiogenesis is essential for malignant tumor growth and metastasis because new vessels offer extra oxygen and nutrients and because endothelial cells in the neovasculature have high permeability.^104^ It was reported that signal transducer and activator of transcription 3 (*STAT3*) activation was capable of increasing *miR-21* expression in exosomes released by CSE-transformed HBECs. In addition, this exosomal *miR-21* could be transferred to normal HBECs and had the ability to increase the levels of *VEGF* in normal HBECs and HUVECs, which promoted angiogenesis in HUVECs. The inhibition of *STAT3* could decrease the levels of exosomal *miR-21* from CSE-transformed HBECs and VEGF in recipient cells, thereby restraining angiogenesis.^105^ Another investigation by Hsu et al.^106^ indicated increased exosome release and *miR-23a* expression in exosomes from lung cancer cells under hypoxic conditions. They also found that exosomal *miR-23a* from hypoxic lung cancer cells could enhance angiogenesis in HUVECs by restraining the expression of prolyl hydroxylase 1 and 2 (*PHD1* and *PHD2*, respectively). Furthermore, downregulating *PHD1* and *PHD2* expression caused the accumulation of *HIF-1α* in endothelial cells, and exosomal *miR-23a* augmented vascular permeability as well as cancer transendothelial migration by suppressing the tight junction protein *ZO-1*. Grimolizzi et al.^100^ also suggested that *miR-126*, which was abundant in exosomes released from NSCLC cells, could induce angiogenesis and malignant transformation in BEAS-2B cells, thereby facilitating the progression of lung cancer, while the suppression of *miR-126* in exosomes by an anti-miR could significantly reverse this effect.

Numerous lines of evidence indicate that EMT may play a key role in the metastasis and invasion of lung cancer.^107^ It was reported that exosomal *miR-193a-3p*, *miR-210-3p*, and *miR-5100* secreted by hypoxic bone marrow-derived mesenchymal stem cells could enhance the invasion and metastasis of lung cancer cells through *STAT3* signaling activation, which induced EMT in these cancer cells.^108^ Some studies have shown that the exosomal miRNA profile of epithelial cells can be altered in the process of EMT.^109^ For example, human LAC A549 cells could release exosomes with increased *miR-23a* expression after mesenchymal transition induced by transforming growth factor-β1 (TGF-β1). Furthermore, it was proven that exosomes derived from epithelial-phenotype A549 cells undergoing EMT could in turn promote mesenchymal transition in other epithelial A549 cells by transferring *miR-23a*.^110^ Rahman et al.^111^ also suggested that exosomes from highly metastatic lung cancer cells and serum from patients with advanced lung cancer could induce EMT in recipient HBECs by carrying vimentin. Nevertheless, this study did not explore how vimentin leads to this effect on HBECs, such as whether exosomal miRNAs act as regulators in this process, since the uptake of specific exosomal miRNAs by epithelial cells can contribute to EMT in these cells as a preceding event. Thus, it is necessary to measure miRNA profiles in exosomes from highly metastatic lung cancer cells and serum from patients with late-stage lung cancer and determine whether there are certain exosomal miRNAs with altered expression as well as the ability to increase vimentin levels in exosomes and cause EMT. A recent study revealed that exosomal *miR-499a-5p* had higher expression in highly metastatic lung cancer cells than in weakly metastatic lung cancer cells and that exosomal *miR-499a-5p* from highly metastatic cancer cells led to EMT in recipient cancer cells by increasing the expression of *p-S6K1* and *p-4E-BP1*, which implied activation of the *MTOR* pathway. An *MTOR* pathway suppressor could weaken the promotive effect on EMT exerted by *miR-499a-5p* mimics.^102^

### Exosomal miRNAs as therapeutic targets in lung cancer

It is well known that resistance to chemotherapeutic drugs accounts for a considerable portion of chemotherapy failure in lung cancer.^112^ Some studies have implied that drug resistance can be transferred from donor cells to recipient cells via exosomes.^113^ One study showed that exosomes from gefitinib-resistant NSCLC cells could contribute to drug resistance acquisition in sensitive NSCLC cells by transferring *miR-214* and that treatment with an exosomal *miR-214* inhibitor could promote the expression of apoptosis-related proteins and the recovery of sensitivity in cancer cells resistant to gefitinib.^114^ Five miRNAs, *miR-197-5p*, *miR-4443*, *miR-642a-3p*, *miR-27b-3p*, and *miR-100-5p*, showed the strongest differential expression in exosomes from cisplatin-resistant lung cancer cells compared with those from cisplatin-sensitive cells.^115^ Further experiments indicated that exosomes from cisplatin-resistant lung cancer cells could increase cisplatin resistance in recipient cells by carrying *miR-100-5p. miR-100-5p* mimics could promote the sensitivity of A549 cells to cisplatin, while *miR-100-5p* inhibitors could facilitate their resistance to chemotherapeutic drugs. With some bioinformatics methods, *mTOR* was predicted as the target gene of *miR-100-5p*. In addition, *miR-100-5p* was confirmed to be negatively correlated with *mTOR* on the basis of analyzing cases from The Cancer Genome Atlas database. However, the experiments did not verify whether exosomal *miR-100-5p* was involved in cisplatin resistance in A549 cells by targeting *mTOR*.^116^ Another investigation also demonstrated that cisplatin resistance was acquired in A549 cells after treatment with exosomes from H1299 cells, which had higher expression of exosomal *miR-96* than A549 cells, and proved that exosomal miR-96 functioned in the acquisition of drug resistance in lung cancer cells by repressing the expression of *LMO7*. In addition, the authors found that fresh A549 cells acquired cisplatin resistance after incubation with exosomes derived from A549 cells that took up exosomes from H1299 cells.^99^ Wei et al.^101^ measured miRNA levels in exosomes released by gemcitabine-resistant lung cancer cells and found that *miR-222-3p* was abundant in these exosomes. Moreover, their results revealed that drug-sensitive lung cancer cells obtained gemcitabine resistance after taking up exosomal *miR-222-3p*, whose target gene was proven to be suppressor of cytokine signaling 3 (*SOCS3*). Evidently, these exosomal miRNAs act as important regulators of drug resistance acquisition in lung cancer cells, which indicates that the turnover of specific miRNAs in exosomes and their target genes may be a new therapeutic strategy for lung cancer.

Radiotherapy is another important therapeutic tool in addition to chemotherapy when lung cancer metastasizes and surgical treatment is not suitable. Radiotherapy resistance can also lead to treatment failure in lung cancer. Zheng et al.^117^ revealed that lung cancer cells treated with X-ray radiation could release *miR-23a* with increased expression in EVs, mainly including exosomes, and the EV *miR-23a* taken up by HUVECs could promote the proliferation and migration of these recipient cells by restraining the expression of phosphatase and tensin homolog (*PTEN*), thereby enhancing angiogenesis and resistance to radiotherapy. They concluded that the EV *miR-23a/PTEN* pathway may act as a new therapeutic target that reduces radiotherapy resistance in lung cancer. YKT6 is a soluble *n*-ethylmaleimide-sensitive factor attachment receptor protein with the function of regulating exosome production and release. One study revealed that YKT6, which was involved in the regulation of these processes, was targeted by *miR-134* and *miR-135b*. In addition, it was discovered that a YKT6 inhibitor could reduce the release of exosomes by 80.9%,^118^ which suggested that the inhibitor has promise as a tool for lung cancer therapy. More broadly, decreasing the production and secretion of exosomes may be an effective treatment for lung cancer.

## Exosomal lncRNAs in lung diseases

Although lncRNAs have been an emerging focus in recent years, there is a lack of published works about the functions of exosomal lncRNAs in other lung diseases beyond lung cancer. Nevertheless, some researchers have revealed that certain lncRNAs also play significant roles in COPD, asthma, ILD, and TB,^119^ which may offer some clues for the further discovery of exosomal lncRNAs that exert effects on the occurrence and development of these lung diseases in the future. Hence, we summarize the roles of only exosomal lncRNAs in lung cancer as the second major component of this review.

### Exosomal lncRNAs in lung cancer

Exosomal lncRNAs can also serve as diagnostic biomarkers in lung cancer. Serum exosomal growth arrest-specific 5 (*GAS5*) was expressed at lower levels in NSCLC patients than in healthy subjects (*p* < 0.001). In addition, patients with advanced-stage NSCLC had lower expression of serum exosomal *GAS5* than did patients with early-stage NSCLC. The ROC curve analysis indicated that the AUC, sensitivity, and specificity of exosomal *GAS5* were 0.857, 85.94%, and 70.00%, respectively, for the diagnosis of NSCLC. The AUC was improved to 0.929 when serum exosomal *GAS5* was combined with the clinical indicator CEA. In addition, this serum biomarker (AUC 0.822, sensitivity 63.16%, specificity 80.00%) also showed better diagnostic power than did CEA (AUC 0.718, sensitivity 50.37%, specificity 85.00%) in detecting early-stage NSCLC.^120^ Another study revealed that plasma exosomal *SOX2-OT* and *ENSG00000245648* were upregulated in plasma exosomes from lung squamous cell carcinoma (LSCC) patients compared with those from negative controls. Only plasma exosomal *SOX2-OT* was significantly downregulated after surgery, which indicated that the levels of exosomal *SOX2-OT* in the plasma could reflect the state of the tumor to some extent. The authors evaluated the diagnostic value of plasma exosomal *SOX2-OT* in distinguishing LSCC from non-LSCC and reported that its AUC, sensitivity, and specificity were 0.815, 76%, and 73.17%, respectively.^121^ Similarly, a study reported that serum exosomal lncRNA *MALAT-1* was more highly expressed in NSCLC patients than in healthy controls, and ROC curves indicated that the AUC, sensitivity, and specificity were 0.703, 0.601, and 0.809, respectively, when serum exosomal *MALAT-1* was tested as a diagnostic biomarker for NSCLC.^122^

Recent studies have demonstrated that lncRNAs in exosomes can play crucial roles in the growth, metastasis, and invasion of lung cancer cells (Table 4). Zhang et al.^122^ found that the levels of *MALAT-1* in serum-derived exosomes were positively correlated with tumor stage and lymphatic metastasis. According to their results, *MALAT-1* contained in exosomes in the serum could promote the proliferation and migration of lung cancer cells by facilitating cell cycle progression and reducing apoptosis in these cells. The knockdown of *MALAT-1* in NSCLC cells could inhibit the proliferation and migration of these cancer cells. The potential mechanism underlying the roles of *MALAT-1* in cell proliferation in NSCLC cells was further explored, and it was revealed that *MALAT-1* knockdown could induce cell apoptosis and decrease the expression of cyclinD1, cyclinD2, and CDK, which contributed to the extension of G1 phase and the shortening of S phase. Previous studies have suggested that TGF-β can promote metastasis in lung cancer cells by combining with a relevant receptor in the cytomembrane and activating downstream proteins in EMT-related signaling pathways.^123^ An investigation explored a new mechanism by which TGF-β mediated metastasis and invasion in lung cancer cells, namely, TGF-β-treated A549 cells secreted exosomes with upregulated lnc-matrix metalloproteinase 2-2 (*lnc-MMP2-2*) expression, which promoted migration and invasion in other A549 cells, as well as permeability in vascular endothelial cells by increasing *MMP2* expression. In fact, the study discovered that the overexpression of *lnc-MMP2-2* could augment vimentin and N-cadherin expression and decrease E-cadherin expression in A549 cells and reduce the tight junction protein levels between vascular endothelial cells, indicating that EMT occurred in recipient A549 cells and led to enhanced blood vessel permeability.^30^ An animal experiment by Cheng et al.^124^ revealed the roles of the exosomal lncRNA *GAS5* in the mechanism of angiogenesis in lung cancer, and the authors found that exosomal *GAS5* was expressed at lower levels in serum from mice with lung cancer than in serum from healthy controls. In addition, exosomes derived from lung cancer cells had the ability to boost the proliferation and tube formation of HUVECs and restrain their apoptosis. Lung cancer cells transfected with *GAS5* overexpression vectors released higher levels of exosomal *GAS5* than the negative control group. In addition, exosomes derived from lung cancer cells overexpressing *GAS5* suppressed cell proliferation and tube formation in HUVECs, in contrast to exosomes from the negative control group. Moreover, the authors showed that *GAS5* had the ability to bind to *miR-29-3p* comparatively with *PTEN* and thereby reduced the expression of *PTEN*, whose downregulation could increase the levels of *p-PI3K* and *p-AKT*. However, it is still not clear whether the function of exosomal *GAS5* in angiogenesis is based on the regulation of *PTEN* expression. Since exosomal lncRNAs participate in the progression of lung cancer, as mentioned above, exosomal lncRNAs have the potential to act as diagnostic biomarkers of lung cancer, and controlling the production and release of exosomes and downregulating the lncRNA levels in exosomes may become novel therapeutic tools for lung cancer treatment in the future.

**Table 4:** The roles of exosomal lncRNAs in the proliferation, metastasis, invasion, and drug resistance of lung cancer cells

Groups	Sample	Exosomal lncRNA	Expression in lung cancer	Functions	References
NSCLC vs. HC	Serum	MALAT-1	Upregulation	Promote proliferation, migration, and reduce apoptosis of lung cancer cell	Zhang et al.^122^
TGF-β-treated vs. non-TGF-β-treated A549 cells	Cell culture	lnc-MMP2-2	Upregulation	Promote metastasis, invasion of cancer cells, and reduce permeability of vascular endothelial cells	Wu et al.^30^
Erlotinib-resistant vs. sensitive NSCLC patients/cells	Serum, cell culture	RP11-838N2.4	Upregulation	Promote erlotinib resistance acquisition of lung cancer cells	Zhang et al.^128^
Gefitinib-resistant vs. sensitive NSCLC cells	Cell culture	lncRNA H19	Upregulation	Promote gefitinib resistance acquisition of NSCLC cells	Lei et al.^129^
Lung cancer vs. healthy mice/cells	Serum, cell culture	GAS5	Downregulation	Promote angiogenesis in lung cancer	Cheng et al.^124^

Some studies have indicated that exosomal lncRNAs are also associated with drug resistance in several kinds of cancer,^125–127^ such as lung cancer. An example can be taken from a study that found increased expression of the lncRNA *RP11-838N2.4* in both NSCLC cells and serum exosomes from NSCLC subjects with erlotinib resistance. In addition, it was proven that the uptake of exosomal *RP11-838N2.4* by sensitive lung cancer cells could lead to erlotinib resistance acquisition in these recipient cells, while knocking out the lncRNA *RP11-838N2.4* could eliminate this effect. Moreover, the lncRNA *RP11-838N2.4* was found to be suppressed by *FOXO1* in NSCLC cells with erlotinib resistance. Nevertheless, a more detailed mechanism has not been elucidated and needs to be further explored.^128^ These findings are in agreement with those of another study that confirmed that the lncRNA *H19* encapsulated in exosomes could induce gefitinib resistance acquisition in NSCLC cells when taken up by these cells. In more detail, the uptake of *H19* packaged in exosomes by lung cancer cells could result in the upregulation of *H19* expression in these recipient cells and thereby cause cancer cell resistance to gefitinib, but *H19* knockdown could contribute to the recovery of gefitinib sensitivity. Furthermore, the packaging of H19 into exosomes was revealed to be mediated by heterogeneous nuclear ribonucleoprotein A2B1 (*hnRNPA2B1*).^129^ The key role of exosomal lncRNAs in drug resistance acquisition by lung cancer cells may offer a clue for approaches aiming to reduce the possibility of chemotherapy failure in lung cancer. In other words, downregulating the expression of lncRNAs in exosomes that promote drug resistance can be beneficial to maintain or recover the sensitivity of cancer cells to chemotherapeutic drugs.

## Potential problems and future directions

Currently, there is much work to be done before exosomal miRNAs and lncRNAs are applied in clinical diagnosis and therapy due to some practical problems.

There are a few obstacles to the application of exosomal miRNAs as diagnostic biomarkers. First, the existing techniques for exosome isolation have a number of shortcomings, which makes it difficult to popularize the use of exosomal miRNAs and lncRNAs as diagnostic biomarkers. The traditional technique, ultracentrifugation, is time consuming and labor intensive despite its consistent yields. Some commercial reagents can simplify the process of exosome extraction, but they are often too expensive to be widely used in clinical diagnosis. In addition, a large number of investigations have discovered various exosomal miRNAs or lncRNAs in specific diseases, but there is still no consensus on the optimal panel of exosomal miRNAs or lncRNAs to use as diagnostic markers for a specific lung disease. In addition, the screening and development of the optimal panel require extensive and accurate studies involving many samples, which is time consuming and labor intensive.

With regard to clinical therapy, there are several challenges to overcome before exosomal miRNAs or lncRNAs can serve as therapeutic targets. First, the targeted drugs that regulate the expression of the crucial RNA should not cause any severe adverse reactions and bind with the specific sites that play critical roles in the pathogenesis of diseases. Despite some advances in RNA-targeted drugs, only one type of synthetic antibiotic has been used in clinical practice, namely, linezolid antibiotics that target RNA with the structure of multihelix junctions.^130^ Therefore, the lack of officially approved miRNA- and lncRNA-targeted drugs is a significant factor that impedes the clinical application of exosomal miRNAs or lncRNAs as therapeutic targets. Furthermore, exosomes used for targeted therapy should be self-derived to avoid the recognition and removal of therapeutic exosomes by the individual’s own immune system. As mentioned above, current techniques for exosome isolation make it time consuming or expensive to produce a large number of exosomes for targeted therapy. Thus, it is an urgent problem and efficient techniques need to be identified for exosome extraction. To increase the rate of specific binding, exosomes should be modified with molecules that have high affinity for the targeted tissues.

## Conclusion

In summary, exosomal miRNAs and lncRNAs mediate cell-to-cell communication, which participates in the initiation and development of several lung diseases, especially lung cancer, through specific mechanisms. The findings stated above can provide some original ideas about how to improve the convenience and efficiency of diagnosing and treating these respiratory disorders in the future. That is, exosomal miRNAs and lncRNAs may act as diagnostic biomarkers and therapeutic targets in lung diseases. Currently, advances concerning the roles of exosomal miRNAs and lncRNAs in lung diseases are limited to only basic research. There are almost no relevant studies reporting that these research results have been applied to the discovery of new drugs in clinical trials or to clinical therapy. However, exosomal miRNAs and lncRNAs have the potential to become diagnostic biomarkers and therapeutic targets in the clinic after the development of technologies involving exosomes and molecular targeted therapy.
